# Biological efficacy of low versus medium dose aspirin after coronary surgery: results from a randomized trial [NCT00262275]

**DOI:** 10.1186/1741-7015-4-12

**Published:** 2006-05-22

**Authors:** Eric Lim, Jacqueline Cornelissen, Tom Routledge, Ayyaz Ali, Stephen Kirtland, Linda Sharples, Kate Sheridan, Sarah Bellm, Helen Munday, Stephen Large

**Affiliations:** 1 Departments of Cardiothoracic Surgery and Clinical Pharmacology, Papworth Hospital, Cambridge, CB3 8RE, UK; 2 Medical Research Council Biostatistics Unit, Cambridge, UK

## Abstract

**Background:**

The beneficial effect of aspirin after coronary surgery is established; however, a recent study reported the inability of low doses (100 mg) to inhibit postoperative platelet function. We conducted a double-blind randomised trial to establish the efficacy of low dose aspirin and to compare it against medium dose aspirin.

**Methods:**

Patients undergoing coronary surgery were invited to participate and consenting patients were randomised to 100 mg or 325 mg of aspirin daily for 5 days. Our primary outcome was the difference in platelet aggregation (day 5 – baseline) using 1 μg/ml of collagen. Secondary outcomes were differences in EC50 of collagen, ADP and epinephrine (assessed using the technique of Born).

**Results:**

From September 2002 to April 2004, 72 patients were randomised; 3 patients discontinued, leaving 35 and 34 in the low and medium dose aspirin arms respectively. The mean aggregation (using 1.1 μg/ml of collagen) was reduced in both the medium and low dose aspirin arms by 37% and 36% respectively. The baseline adjusted difference (low – medium) was 6% (95% CI -3 to 14; p = 0.19). The directions of the results for the differences in EC50 (low – medium) were consistent for collagen, ADP and epinephrine at -0.07 (-0.53 to 0.40), -0.08 (-0.28 to 0.11) and -4.41 (-10.56 to 1.72) respectively, but none were statistically significant.

**Conclusion:**

Contrary to recent findings, low dose aspirin is effective and medium dose aspirin did not prove superior for inhibiting platelet aggregation after coronary surgery.

## Background

Normal platelet physiology cannot be assumed to prevail in conditions of severe physiological stress such as major surgery. However, the impact of antiplatelet therapy is critical in the early postoperative period after coronary surgery as it confers lower in-hospital mortality [1] and morbidity and improves graft patency [2].

The motivation for the present study was a report that low dose aspirin (100 mg) did not inhibit collagen-induced platelet aggregation after cardiac surgery [3]. Moreover, our systematic review and indirect comparison meta-analysis suggested that trials using medium dose aspirin regimens (325 mg) after coronary surgery could have better graft patency rates than trials using low dose aspirin (75 to 150 mg) [4]. The evidence from both these sources suggests that equivalence cannot be assumed between the two dosing regimens.

To evaluate the biological efficacy of low dose aspirin and to compare it against a medium dose preparation, we conducted a double-blind randomized trial of low against medium dose aspirin after coronary artery bypass surgery.

## Methods

### Participants

We conducted a Local Research Ethics Committee (Huntingdon Local Ethics Research Committee) approved prospective randomised trial. All patients undergoing elective primary coronary artery bypass surgery were invited to participate, and informed consent was obtained. We excluded patients if they did not stop antiplatelet therapy a week prior to surgery, had contraindications to aspirin or were on other medications that interact with aspirin, if surgery was performed without cardiopulmonary bypass, if platelet transfusion was administered intra-operatively or within the first 24 hours and if extubation was not achieved in the first 24 hours (to ensure all patients had 5 full days of oral antiplatelet therapy).

### Intervention and randomisation

On the first postoperative morning, patients were randomised to receive one of the following identically encapsulated treatments: aspirin 100 mg or aspirin 325 mg for 5 days. Randomisation was undertaken by pharmacy into treatment allocation blocks of 6 and medications were stored in numbered containers. Participants, researchers and statisticians were blind to the treatment allocations. All patients routinely received low molecular weight heparin postoperatively.

### Outcome measures

Our primary outcome measure was percentage aggregation on day 5 (2 hours after drug administration,) expressed as percentage of baseline, using 1.1 μg/ml collagen as an agonist. Assessment of platelet aggregation was undertaken using the technique of Born [5]. Secondary outcome measures were the effective concentrations of Horm collagen, adenosine diphosphate (ADP) and epinephrine on day 5 required to produce 50% aggregation (EC50) compared to baseline.

### Laboratory methods

Venous blood (30 ml) was collected into 3.8% trisodium citrate monovettes (Sarstedt) and gently inverted to ensure mixing. Within 30 minutes of venepuncture, samples were centrifuged at 1000 r.p.m for 15 minutes to obtain platelet rich plasma (PRP). Platelet poor plasma (PPP) was prepared by centrifuging 1 ml of PRP at 6000 r.p.m. for 1 minute. Platelet aggregation was determined turbidimetrically using the Platelet Aggregation Profiler^® ^PAP-4 (BioData Corporation, PA, USA), with baseline optical density set with PPP. PRP samples (225 μl) were pre-warmed to 37°C for 30 seconds before the addition of agonist (25 μl), with the stir bar rate set at 1000 r.p.m. PRP samples were stimulated for 4.5 minutes with freshly prepared adenosine 5'-diphosphate (ADP, Sigma; final concentration range of 0.25–5.0 μmol/l), Horm collagen (Axis-Shield Diagnostics; range 0.11–4.4 μg/ml) and epinephrine (Sigma; range 0.125–5.0 μmol/l). Stock saline solutions of epinephrine and ADP were stored at -80°C, with appropriate precautions taken to prevent the light-dependent degradation of epinephrine. Platelet aggregometry readings for each agonist were converted to EC50 using curve fit software. The EC50 represents the concentration of agonist required to cause 50% of maximal aggregation.

### Sample size

The original trial started as a three arm study (low dose aspirin, medium dose aspirin and clopidogrel) intending to recruit 108 patients with 36 participants in each arm. We aimed to be able to detect a difference of 30% in post-operative platelet aggregation at day 5, expressed as a percentage of pre-operative values between any two arms.

An interim analysis of 54 patients (18 in each arm) was planned with the stopping criterion of a significant difference at 2% level. At that stage, there would be at least 90% power to detect a difference of at least 1.5 standard deviations, and the inferior arm would be terminated.

### Statistical methods

Patients were grouped according to treatment allocation. Categorical data are presented as frequency (%) and continuous data as mean with standard deviation (SD) or median with interquartile range (IQR). Comparisons of categorical data between groups were made using Fisher's exact test or Pearson's Chi Squared test. Continuous data were compared using Student's t-test or Wilcoxon rank sum as appropriate. Primary outcome was compared using regression analysis (ANCOVA) with robust standard errors to adjust for baseline values of platelet aggregation and EC50 concentrations. Analyses were performed using Stata 8.2 (StataCorp, Texas, USA).

## Results

From September 2002 to April 2004, patients were consecutively screened for eligibility and 200 participants were invited to participate, of whom 116 consented. A further 26 did not meet the inclusion criteria, leaving 90 patients suitable for randomization. At interim analysis, after 54 were randomized (18 in each arm), and the clopidogrel arm was terminated in accordance to pre-planned criterion owing to concerns for participant safety [6].

Of the 90 patients, a total of 36 were randomized to medium dose aspirin, 36 to low dose aspirin and 18 to clopidogrel. One patient did not receive allocated medication and another withdrew consent to the study in the low dose aspirin arm. One patient did not receive the allocated medication and 2 were withdrawn (abdominal pain, re-operation) in the medium dose aspirin arm. We analyzed the results from 35 and 34 patients in the low and medium dose arms respectively by intention-to-treat. As the results for patients in the clopidogrel arm have been published [6], we report the results of the patients in the two aspirin arms.

### Primary outcome

The patients were well matched for baseline characteristics (Table 1). Mean platelet aggregation (using 1.1 μg/ml of collagen) was reduced in both medium and low dose aspirin arms by 37% and 36% respectively (table 2). The baseline adjusted difference comparing low to medium dose (low – medium dose) was 6% (95% CI -3 to 14; p = 0.19) in favour of medium dose but not statistically significant.

### Secondary outcomes

The directions of the results for the differences in EC50 concentrations were consistent for all three agonists in favour of medium dose, although none were statistically significant. The baseline adjusted EC50 concentrations (low – medium dose) for collagen, ADP and epinephrine were -0.07 μg/ml (-0.53 to 0.40), -0.08 μmol/l (-0.28 to 0.11) and -4.41 μmol/l (-10.56 to 1.72) respectively.

## Discussion

The results of our study confirm that both aspirin doses (325 mg and 100 mg) were effective in the inhibition of platelet aggregation after cardiac surgery. The mean reductions by day 5 compared to baseline were 37% and 36% for the medium and low dose aspirin regimens respectively.

### Efficacy of low dose regimen

Our results contradict the findings of the observational study by Zimmerman [3], who reported that 100 mg of aspirin was insufficient to inhibit platelet aggregation after coronary surgery, with a mean aggregation of 103% of the baseline value in his series. It is not possible to determine why the results for low dose aspirin from our centre in Cambridge were so different from those of Zimmerman in Düsseldorf. The baseline characteristics of our patients were comparable, and our laboratory methods were similar. However, unmeasured differences can exist. A potential difference could be in the genetic makeup with respect to aspirin resistance. The prevalence of Pl^A2 ^polymorphism (linked to aspirin resistance) has been estimated at 39% in patients with ischaemic heart disease [7], and has greater potential to influence the results of studies with smaller sample sizes. The total number of participants in the observational series by Zimmerman was 24.

### Clinical implications

Throughout, we have used platelet aggregation as a surrogate for clinical outcome, as clinical efficacy cannot be assumed without evidence of inhibition. Our previous indirect comparison meta-analysis suggested that the efficacy of low dose aspirin cannot be assumed to be equivalent to superior results with medium dose regimens in the preservation of graft patency [4]. In vitro, however, medium dose aspirin did not prove superior in the inhibition of platelet aggregation. The upper limit of the 95% confidence interval was 14% for the primary outcome measure in favour of medium dose aspirin, and it is difficult on the limited information available to be certain if this excludes a clinically meaningful or important difference. Unfortunately (as in most research) we have generated more questions rather than clarifying answers. Further translational research evaluating (in part) the effects of platelet aggregation on clinical outcomes would be invaluable.

### Standardization of platelet aggregation

A difficulty that we encountered in comparing our results with those from other centres was the lack of standardization in measuring platelet aggregation. The most common method is to use a fixed concentration of agonist to compare percentage aggregation to baseline. However, the drawbacks of this technique is that some samples do not aggregate with a low dose of agonist or aggregate completely when a high dose is selected. Moreover, as different fixed concentrations are used, it is not possible to compare the results between centres. A solution is to report EC50s, the concentrations of agonist that produce 50% aggregation. In this way, it is possible to avoid the arbitrary selection of a fixed dose of agonist and also to allow results from different studies to be compared.

## Conclusion

Low dose aspirin is effective and medium dose aspirin did not prove superior as an inhibitor of platelet aggregation in vitro after coronary surgery. Further studies are required to compare clinical outcomes with platelet aggregation results, and researchers should consider the use of EC50 to report the results of platelet inhibition to facilitate comparisons among different centres.

## Competing interests

The author(s) declare that they have no competing interests.

## Authors' contributions

EL conceived and designed the study, performed the statistical analysis and drafted the initial and final manuscript; JC was involved in designing the study, performed the aggregometry, coordinated the clinical trials and approved the final manuscript; TR and AA were involved in coordinating the clinical trials and data collection and helped draft the initial manuscript; SK was involved in designing the study and approved the final manuscript; LS helped design the study, performed the statistical analyses and approved the final manuscript; KS and SB performed aggregometry, helped in data collection and approved the final manuscript; HM was involved in coordinating the clinical trials and approved the final manuscript; SL was the overall study supervisor, who participated in the design, conduct and management of the project and approved the final manuscript.

## Pre-publication history

The pre-publication history for this paper can be accessed here:



## Figures and Tables

**Figure 1:**
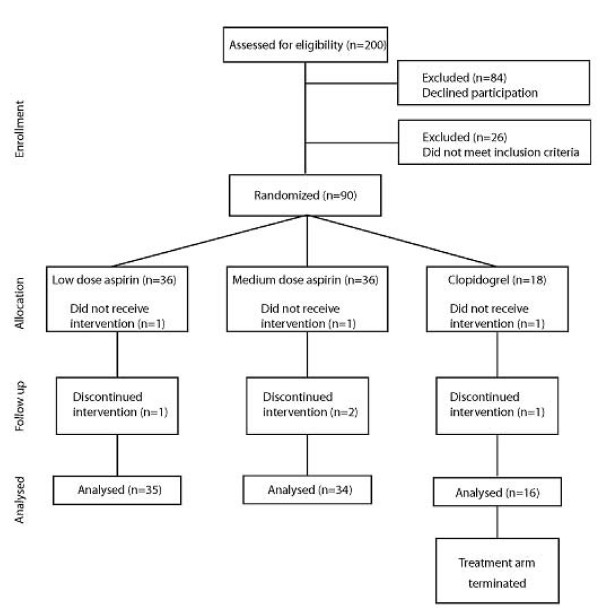
Trial flow diagram. *Footnote*: *numbers that were analyzed were more than expected, taking into account patients who discontinued or did not receive allocated medication owing to intention-to-treat analysis*.

**Table 1:** Baseline characteristics of patients randomised to aspirin

	Medium dose	Low dose
Sample size	36	36
Mean age, years (SD)	66 (6)	66 (8)
Mean height, m (SD)	1.75 (0.08)	1.73 (0.07)
Mean weight, kg (SD)	83.1 (2.4)	85.3 (2.6)
Male sex, n (%)	33 (92)	33 (92)
Mean platelet count, × 10^9 ^(SD)	229 (59)	211 (66)
		
Diabetes, n (%)	4 (11)	10 (28)
Hypercholesterolaemia, n (%)	33 (92)	32 (89)
Hypertension, n (%)	21 (58)	24 (67)
Current smokers, n (%)	3 (8)	0 (0)
		
Median number of grafts, n (IQR)	4 (3 to 4)	3.5 (3 to 4)
Median bypass time, min (IQR)	73 (60 to 86)	70 (54 to 87)

**Table 2:** Effects of aspirin on collagen induced platelet aggregation and EC50 concentrations postoperatively (final – baseline)

	Baseline (SD)	Final (SD)	Difference (95% CI)	P value
*Percentage aggregation (using 1.1 μg/ml collagen)*				
Medium dose	70 (28)	32 (15)	-37 (-46 to -28)	<0.001
Low dose	76 (21)	39 (21)	-36 (-46 to -27)	<0.001
*Mean EC50 concentration (using 0.11 – 4.4 μg/ml collagen)*				
Medium dose	0.53 (0.58)	1.79 (0.91)	1.23 (0.95 to 1.52)	<0.001
Low dose	0.48 (0.51)	1.69 (1.10)	1.20 (0.80 to 1.59)	<0.001

**Table 3:** Adjusted outcome measures comparing low to medium dose aspirin (low – medium)

	Baseline adjusted difference (95% CI)	P value
Change in percentage aggregation *		
Collagen 1 μg/ml	6 (-3 to 14)	0.19
ADP 5 μmol/l	6 (0 to 13)	0.07
Epinephrine 10 μmol/l	3 (-5 to 11)	0.44
		
Change in EC50 concentration **		
Collagen μg/ml	-0.07 (-0.53 to 0.40)	0.77
ADP μmol/l	-0.08 (-0.28 to 0.11)	0.40
Epinephrine μmol/l	-4.41 (-10.56 to 1.72)	

* Positive results favour greater treatment effect for medium dose aspirin

** Negative results favour greater treatment effect for medium dose aspirin
